# Liver Transcriptome Analysis Reveals the Mechanisms of Metabolic Adaptation of Xizang Sheep to Seasonal Changes

**DOI:** 10.3390/metabo14110640

**Published:** 2024-11-19

**Authors:** Jianzhao Cui, Junru Pan, Fengbo Sun, Nan Zhang, Jiacuo Jinmei, Yang Zhen, Ciren Puchi, Luo Snag, Zengqiang Liu, Wangsheng Zhao, Yangzong Zhaxi

**Affiliations:** 1 Institute of Animal Science, Xizang Academy of Agricultural and Animal Husbandry Science, Lhasa 850009, China; rkzswkj@163.com (J.C.); 18076993666@163.com (Z.L.); 2 Shigatse Science and Technology Bureau, Shigatse 857000, China; 3 Key Laboratory of Animal Genetics and Breeding on Xizang Plateau, Ministry of Agriculture and Rural Affairs, Lhasa 850009, China; 4 School of Life Sciences and Engineering, Southwest University of Science and Technology, Mianyang 621000, China; panjunru5@163.com; 5 Xizang Autonomous Region Center for Disease Control and Prevention, Lhasa 850009, China; 18289146929@163.com (F.S.); nanan_cc2008@126.com (N.Z.); xmk22295@163.com (J.J.); 13908988062@163.com (Y.Z.); 6 Agricultural and Rural Bureau of Jiangzi County, Jiangzi 857400, China; 18184992208@163.com (C.P.); 13889022111@163.com (L.S.)

**Keywords:** Xizang sheep, liver, seasonal changes, transcriptome, metabolic adaptation

## Abstract

**Background/Objectives**: The Xizang sheep is a unique breed of sheep in the highland regions of China that has gradually developed physiological characteristics adapted to the plate environment through long-term natural selection and artificial breeding. However, little is known about the molecular basis of metabolic adaptation to seasons in Xizang sheep. **Methods**: In this study, liver tissues from Xizang sheep in summer (SL) and autumn (AL) were selected for transcriptome sequencing to explore the metabolic adaptability of Xizang sheep to seasons. **Results**: The results showed that a total of 12,046 differentially expressed genes (DEGs) were identified, with 1123 genes significantly upregulated and 951 genes significantly downregulated in autumn. The top five pathways enriched for DEGs were Metabolic pathways, Phagosome, Valine, leucine and isoleucine degradation, Propanoate metabolism, and Fatty acid metabolism, which are involved in immune regulation, fat oxidation, and synthesis. The reduction in lipid synthesis, fatty acid oxidation, and fat breakdown metabolism promotes gluconeogenesis by inhibiting the Peroxisome proliferator-activated receptors (PPAR) and Phosphoinositide 3-kinase- Protein kinase B (PI3K-Akt) signaling pathways. **Conclusions**: This process helps to maintain the whole-body energy homeostasis of Xizang sheep, facilitating their adaptation to the seasonal changes in the extreme high-altitude environment. These findings provide foundational data for studying the molecular mechanisms of metabolic adaptation to seasons in ruminants.

## 1. Introduction

The Qinghai-Tibet Plateau, known as the “Third Pole” on Earth due to its unique extreme environmental conditions, including low oxygen levels, high ultraviolet radiation, and low temperatures, poses a severe challenge to the survival of many species [1]. However, the Xizang people and the wild and domestic animals living on the plateau have adapted to these hypoxic environments, developing unique lifestyles, dietary habits, and genetic characteristics [2,3,4,5]. Currently, numerous studies have revealed the potential mechanisms of environmental adaptation in plateau animals (such as pigs, cattle, and sheep) [6,7,8]. Nevertheless, the specific mechanisms by which plateau animals precisely adapt to the plateau environment still require further in-depth research and exploration.

Xizang sheep (*Ovis aries*), as one of the main domestic animals in the Qinghai-Tibet Plateau region, are significantly affected by seasonal changes in their survival and production performance [9,10]. As a key metabolic organ in animals, the liver is responsible for various functions such as energy metabolism, detoxification, and protein synthesis, and changes in its transcriptome can directly reflect the animal’s adaptability to environmental changes [11,12,13]. Cao et al. used transcriptome technology to study the differences in gene expression in the livers of Xizang chickens and other lowland chicken breeds during hypoxia adaptation, finding that several differentially expressed genes (DEGs) are widely involved in lipid metabolism processes, such as Carnitine O-palmitoyltransferase 1 Alpha (*CPT1A*), Peroxisome Proliferator-Activated Receptor Gamma, Coactivator 1 Alpha (*PPARC1A*), Stearoyl-CoA Desaturase (*SCD*), and Fatty Acid Synthase (*FASN*) [14]. Feng et al. compared the miRNA transcriptomes of six hypoxia-sensitive tissues (heart, kidney, liver, lung, skeletal muscle, and spleen) in goats from different altitudes (600 m and 3000 m), revealing the post-transcriptional regulation of the hypoxia inducible factor 1, insulin, and p53 signaling pathways [15]. Studies have reported that in yaks, genes related to fatty acid uptake (Solute Carrier Family 27 Member 2, *SLC27A2*) and fatty acid oxidation (Carnitine O-Palmitoyltransferase 1C, *CPT1C*) in the liver are downregulated, indicating that fatty acid synthesis is suppressed in yaks during the cold season (winter) [16,17]. Despite this, research on how Sewa Sheep adapt to seasonal changes in the plateau environment remains scarce.

Little is known about how the liver of Sewa Sheep meets different energy demands during seasons of summer and autumn at the mechanistic level. This study aims to analyze the transcriptome characteristics of the liver in grazing Sewa Sheep during different seasons, such as summer and autumn, using high-throughput sequencing technology, to reveal the molecular mechanisms of their metabolic adaptability. By comparing gene expression differences in liver tissues across seasons, we can identify genes and signaling pathways associated with seasonal metabolic changes. This helps to reveal how Sewa Sheep adapt to the seasonal changes in extreme high-altitude environments, especially in terms of energy demands and metabolic pathways, providing theoretical references for the breeding and production management of Xizang sheep.

## 2. Materials and Methods

### 2.1. Animals and Sample Collection

In this study, Xizang sheep (Sewa Sheep) from Bangga County in Xizang were selected as the subjects of research. During the summer and autumn, five healthy ewes with similar body weights and ages were randomly selected from each season. All sheep were grazed on natural grassland at an elevation of roughly 4750 m above sea level. During the experimental period, the sheep had unrestricted access to food and water. The samples were divided into a summer group (SL, *n* = 5) and an autumn group (AL, *n* = 5), with SL serving as the control group and AL as the treatment group. After the sheep were slaughtered, liver tissues were collected. Before RNA extraction, the tissue samples were quickly frozen in liquid nitrogen and stored at −80 °C.

### 2.2. RNA Extraction and cDNA Synthesis

Total RNA was extracted from XIzang sheep liver samples using RNAiso Plus reagent (Takara Biotechnology Co., Ltd., Dalian, China) according to standard methods. Subsequently, mRNA was reverse transcribed into cDNA using the PrimeScript™ RT reagent Kit with gDNA Eraser (Takara).

### 2.3. Mapping of Sequencing Reads and Identification of DEGs

Cutadapt Version 1.2.1 was utilized to eliminate raw reads containing adapters. Only clean reads were utilized for gene expression mapping to the ovine genome assembly version 3.1 (Oar_v3.1) using TopHat v2.0.1.2. To assess gene expression levels across identical samples, DESeq v1.18.0 was applied. The RPKM (Reads Per Kilobase per Million mapped reads) values were calculated by normalizing gene enrichment to the library size and gene length, using the Ensembl gene annotations for Oar_v3.1. Genes were considered expressed if their RPKM exceeded 0.01 in at least two samples per library. DESeq was also used to identify differentially expressed genes (DEGs) with a |log2 fold change| greater than 1 and adjusted *p*-values less than 0.05.

### 2.4. GO and KEGG Pathway Analyses

The GOseq R package (http://kobas.cbi.pku.edu.cn, accessed on 30 September 2024) was used for GO analysis. GO analysis was performed to identify and compare the functionalities of the DEGs in terms of molecular function, biological process, and cellular component. GO terms with corrected *p*-values < 0.05 were considered significantly enriched. The DEGs were compared with the KEGG pathway database using the KOBAS 2.0 program (http://bioinf.wehi.edu.au/software/goseq, accessed on 30 September 2024). KEGG pathway analysis was performed to construct the gene pathway interactions. Pathways with corrected *p*-values < 0.05 were considered highly enriched.

### 2.5. Reverse Transcription-Quantitative PCR Validation of DEGs

Six DEGs were randomly selected for quantitative real-time PCR (qRT-PCR) analysis to validate the accuracy of the RNA-Seq results, using the same RNA samples that were sequenced. cDNA synthesis was performed according to the protocol provided with the Quantscript RT Kit by Tiangen Biotech Co., Ltd. (Beijing, China). Primer sequences, as listed in Table 1, were synthesized by Shanghai Sangon Bioengineering Technology Service Co., Ltd., Shanghai, China. The RT-qPCR was conducted using a CFX96 TouchTM real-time PCR detection system (Bio-Rad, Munich, Germany) and SYBR Green Super-Real Pre-Mix Plus (Tiangen Biotechnology Co., Ltd., Beijing, China). Data analysis was conducted using the 2^−ΔΔCt^ method, and GraphPad Prism 9 software was employed to perform Student’s *t*-tests to assess the expression differences between samples SL and AL.

## 3. Results

### 3.1. Analysis of Gene Differential Expression Between Groups in Different Seasons

A differential expression analysis was conducted between the two groups using DESeq2, with the selection criteria being FDR < 0.05 and |log2Fold Change| >= 1.

**Figure 1:**
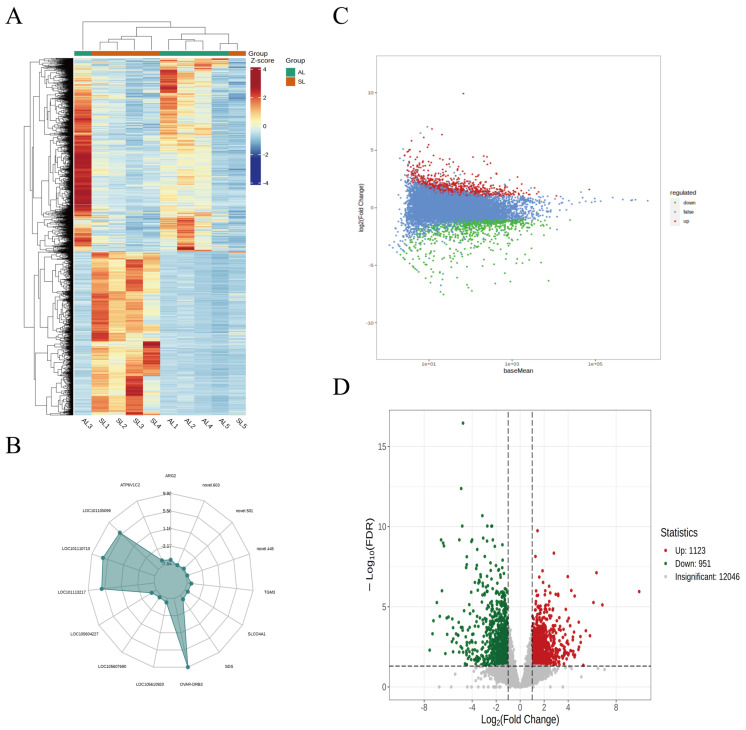
Visualization of DEGs. (**A**) A heatmap displaying differential gene clustering that displays the expression profiles of DEGs across several samples. (**B**) The top 15 differentially expressed genes (downregulated exclusively) are displayed on the DEG radar map. (**C**) The MA plot displays DEGs according to the sample’s log2-fold change (log2FC) values (y-axis) and average expression level (x-axis). Genes that show no appreciable variation are denoted by blue dots, downregulated genes by green, and upregulated genes are indicated by red dots. (**D**) Volcano map. The expression’s fold change is shown on the x-axis, and the modified *p*-value (significance level) is shown on the y-axis. Genes that are upregulated or downregulated are indicated by red and green dots, respectively, while genes that do not differ significantly are indicated by gray dots.

A total of 12,046 differentially expressed genes were identified in the liver tissues of the two groups (Figure 1C,D), including 1123 upregulated DEGs and 951 downregulated DEGs. A radar chart shows the 15 genes with the most significant expression differences; among these, four genes were significantly upregulated, and 11 genes were significantly downregulated (Figure 1B). Cluster analysis revealed that genes with high and low expression levels were clustered separately in the livers of groups SL and AL. (Figure 1A). Table 2 shows the top 10 significantly upregulated genes and the top 10 significantly downregulated genes.

### 3.2. GO Analysis of Differentially Expressed Genes

**Figure 2:**
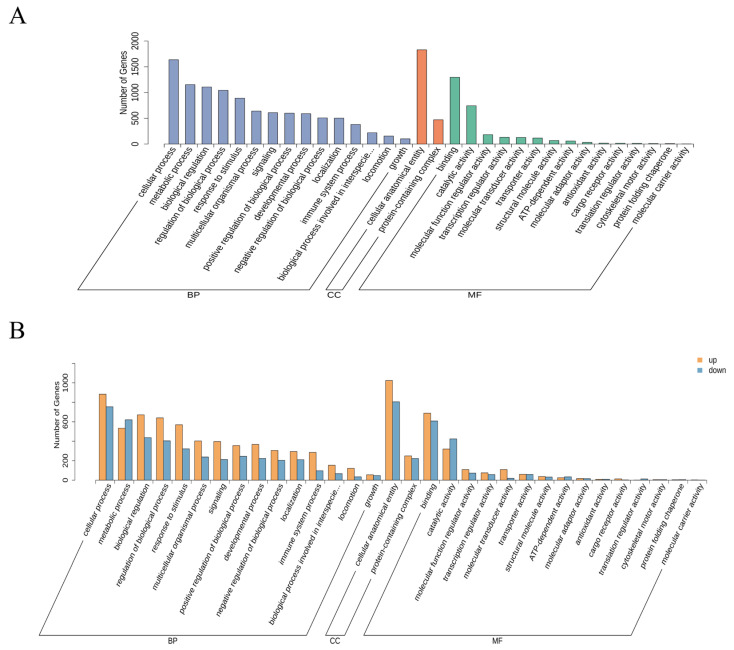
GO annotation analysis of DEGs. (**A**) GO classification histogram showing the distribution of DEGs on different secondary GO terms (x-axis). The number of DEGs connected to each item is displayed on the y-axis. (**B**) Histograms of the up- and downregulation of GO, illustrating patterns of differential expression within a given second-order GO term (x-axis). Each item’s DEG count is displayed on the y-axis, with the yellow bar representing upregulated genes and the blue bar representing downregulated genes.

Group AL and Group SL underwent enrichment analysis and GO functional annotation. A histogram was created, and the top 30 most significantly enriched GO terms in the comparison groups were examined using the three components of molecular function (MF), biological process (BP), and cellular component (CC). The top five GO terms related to BP were cellular process, metabolic process, regulation of biological process, biological regulation, and response to stimulus; the top five GO terms related to MF were binding, catalytic activity, molecular transducer activity, molecular function regulator activity, and transcription regulator activity (Figure 2A). The GO terms related to CC were protein-containing complex and cellular anatomical entity. GO classification statistics were performed for upregulated and downregulated differentially expressed genes (DEGs). The top five GO terms were consistent with the results sorted by the number of genes (Figure 2B).

### 3.3. KEGG Analysis of DEGs

**Figure 3:**
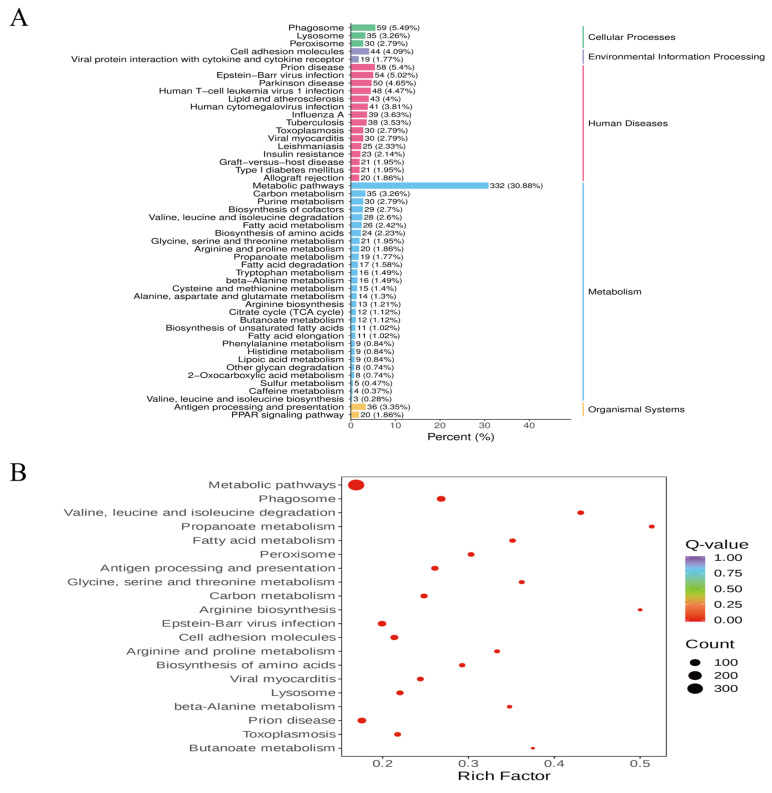
Analysis of KEGG pathway enrichment. (**A**) Bar graph of the enrichment pathway, with the x-axis representing the number of DEGs each KEGG pathway has annotated, and the y-axis showing the pathway name. The number of DEGs mapped to each path is indicated by the bar height, and the numbers enclosed in parentheses represent the path annotation’s DEGs as a percentage of all DEGs. The rightmost label represents the classification of KEGG paths. (**B**) Enrichment scatter plot: the x-axis represents the enrichment factor, and higher values indicate greater enrichment. The KEGG pathway is represented by the y-axis, and the number of DEGs enriched in that pathway is indicated by the size of each point: the larger the point, the more DEGs. The redder the dots, the more significant they are.

**Figure 4:**
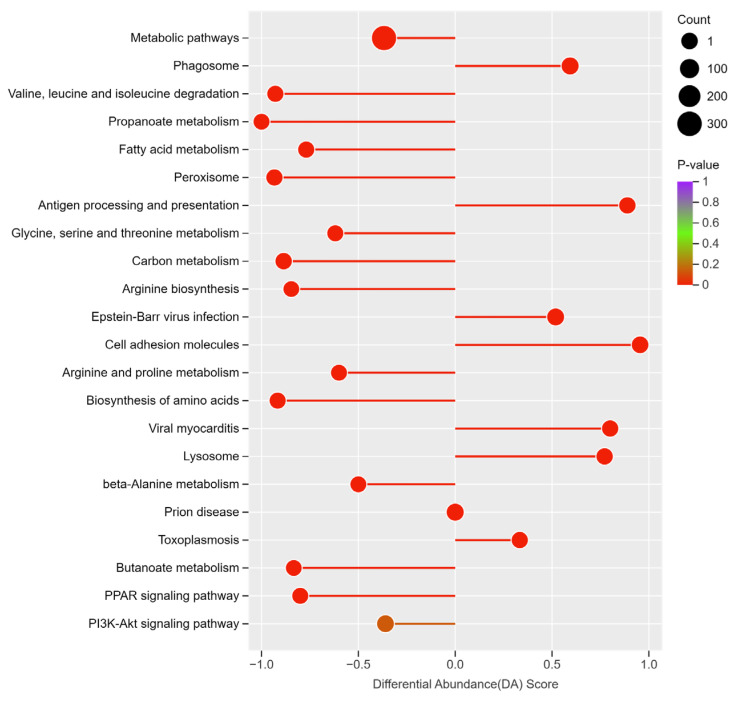
The DA score plot for the overall changes in pathways.

To gain a deeper understanding of the roles of differentially expressed genes (DEGs) in the liver tissue of Xizang sheep across different seasons, a KEGG enrichment analysis was conducted. As shown in Figure 3A, the five main categories of enriched pathways were Metabolism, Cellular Processes, Organismal Systems, Human Diseases, and Environmental Information Processing. A total of 20 pathways were found to be significantly enriched; the top five enriched pathways were Metabolic pathways, Phagosome, Valine, leucine and isoleucine degradation, Propanoate metabolism, and Fatty acid metabolism (Figure 3B). An analysis of the overall changes in 22 important pathways showed that, compared with the SL group, the Metabolic pathways, Valine, leucine and isoleucine degradation, Propanoate metabolism, PI3K-Akt signaling pathway, PPAR signaling pathway, and Fatty acid metabolism were all downregulated in the AL group, while the Phagosome pathway showed an upregulation trend (Figure 4).

### 3.4. KOG Annotation and Classification Statistics of DEGs

**Figure 5:**
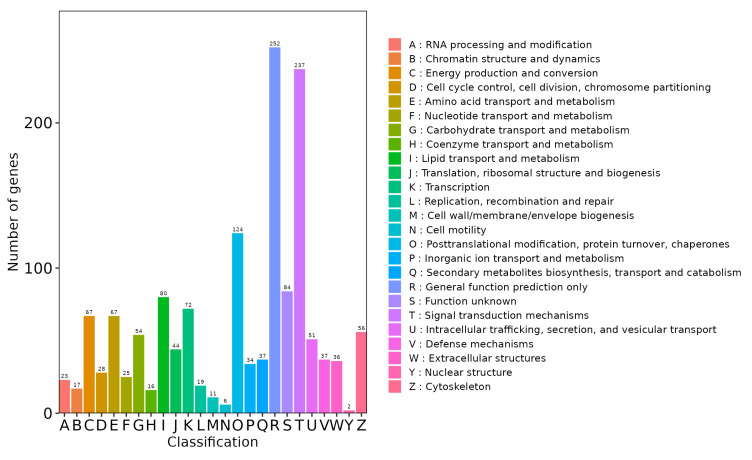
KOG functional classification of DEGs. This bar chart visualizes the distribution of DEGs across different KOG functional categories. The x-axis shows the KOG ID code, and the y-axis represents the number of DEGs associated with each category. The different colors distinguish the KOG class, and the legend provides the KOG ID code and its corresponding functional description.

KOG refers to the clustering of orthologous proteins from eukaryotes to annotate eukaryotic genes. As shown in Figure 5, the differentially expressed genes (DEGs) were categorized into 25 KOG clusters, with the largest proportion of matched proteins in the “General function prediction only” category among all KOGs.

### 3.5. qPCR Validation

**Figure 6:**
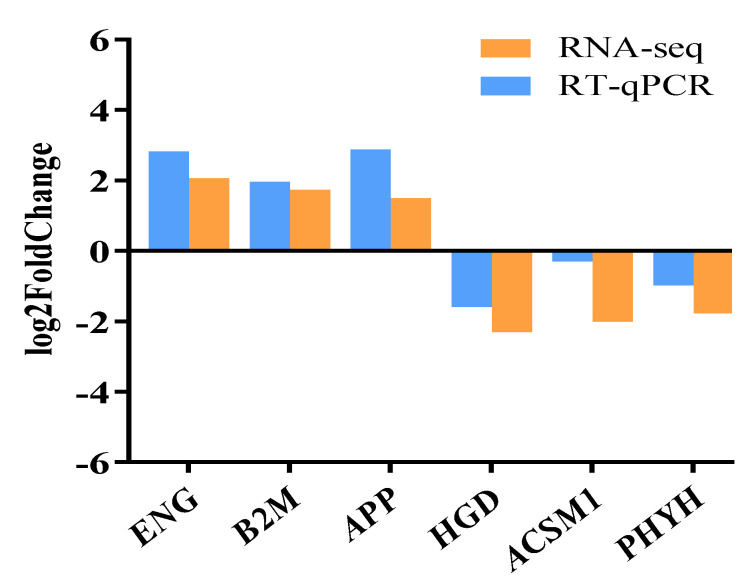
DEG’s RNA-Seq and RT-qPCR results are compared. A subset of genes discovered by RNA-Seq and RT-qPCR had their relative expression levels compared. The value is expressed as a Log2-fold change (FC), which indicates the relative abundance of each gene, and the RT-qPCR data normalized to the expression level of the *GAPDH* gene.

To validate the RNA sequencing results, we conducted qPCR validation on six randomly selected DEGs, which were *ENG*, *B2M*, *APP*, *HGD*, *ACSM1*, and *PHYH*. Notably, the qPCR results showed significant consistency with the RNA sequencing data (Figure 6).

## 4. Discussion

This study reveals the potential mechanisms underlying the seasonal adaptability of Sewa sheep, which adjust gene pathways related to liver energy metabolism and immune function to minimize fat breakdown and maintain whole-body energy homeostasis, further adapting to the seasonal changes of the Qinghai-Tibet Plateau.

Ureña et al. compared the transcriptomes of ram semen in summer and autumn, assessing 236 significantly differentially expressed genes (DEGs), with the majority (228) being downregulated [18]. The main functions of these genes are related to sexual reproduction, protein metabolic processes, and negative regulation of kinase activity [18]. A study analyzing the liver transcriptome sequencing of Hu sheep and Tibetan sheep found that Acyl-CoA Synthetase Long-Chain Family Member 1 (*ACSL1*), Acyl-CoA Synthetase Long-Chain Family Member 4 (*ACSL4*), Acyl-CoA Synthetase Long-Chain Family Member 5 (*ACSL5*), Carnitine O-Palmitoyltransferase 1A (*CPT1A*), *CPT1C*, Solute Carrier Family 25 Member 20 (*SLC25A20*), and Fibroblast Growth Factor 21 (*FGF21*) are involved in the host’s adaptation to high-altitude environments [19]. Pan et al., by comparing the rumen transcriptomes of high-altitude Xizang goats and low-altitude goats, found that genes like Solute Carrier Family 26 Member 9 (*SLC26A9*), Glutathione Peroxidase 3 (*GPX3*), Arrestin Domain Containing 4 (*ARRDC4*), and Cytochrome c Oxidase Subunit 1 (*COX1*) may be involved in the adaptation of Xizang goats to the extreme climate of high altitudes [20]. Therefore, the host transcriptome plays a significant role in adapting to climatic environments. In this study, a total of 12,046 DEGs were identified in the two groups of liver tissues, with 1123 upregulated and 951 downregulated; the top five pathways enriched for these DEGs were Metabolic pathways, Phagosome, Valine, leucine and isoleucine degradation, Propanoate metabolism, and Fatty acid metabolism. In the Phagosome pathway, most genes were significantly upregulated in the autumn, including DQ Alpha (*DQA*), Integrin Beta 5 (*ITGB5*), Calnexin (*CANX*), and ATPase, H+ Transporting, Lysosomal Accessory Protein 1 (*ATP6AP1*), among others. Studies have shown that *DQA*, located in the MHC IIa class region, controls the self/non-self recognition of the immune system and plays an important role in immune responses to adaptive traits [21]. *ITGB5* encodes the integrin β5 subunit, which combines with the αv subunit to form integrin αvβ5, a complex that plays a role in the innate defense system and can fend off bacteria [22]. The *CANX* gene is associated with immune regulation, and its expression significantly enhances the in vitro immune attack mediated by CD8 T cells [23,24]. *ATP6AP1,* by participating in immune regulation, may function as a tumor suppressor gene [25]. Therefore, this study speculates that the upregulation of immune-related genes (*DQA*, *CANX*, *ATP6AP1* and *ITGB5*) in the liver of Xizang sheep in autumn is beneficial for enhancing their immunity to adapt to the environment. In the valine, leucine, and isoleucine degradation pathway, we identified a total of 28 enriched genes, with 27 genes significantly downregulated in autumn, including Branched Chain Keto Acid Dehydrogenase E1, Beta Polypeptide (*BCKDHB*), *BCKDHA* and Branched Chain Amino Acid Transaminase 2 (*BCAT2*). In BCAA metabolism, BCAT2, as a branched-chain aminotransferase (BCAT), promotes the transamination of BCAAs to branched-chain α-keto acids (BCKAs), such as α-KIC, α-KMV, and α-KIV [26]. BCKDHB, together with other subunits (primarily BCKDHA), forms the branched-chain keto acid dehydrogenase complex (BCKDC), which catalyzes the dehydrogenation of branched-chain α-keto acids (BCKAs) to produce the corresponding branched-chain acyl-CoAs. This further influences the metabolism of branched-chain amino acids (BCAAs) [27,28]. Valine, leucine, and isoleucine are three essential amino acids that cannot be synthesized by animals and must be obtained through diet [29]. Compared to summer, the nutritional value of pasture in autumn is lower, with characteristics such as lower dry matter content and reduced crude protein levels [30]. These findings suggest that the expression of genes involved in the valine, leucine, and isoleucine degradation pathway may be regulated by the nutritional components of the forage. Previous studies have shown that supplementary feeding can improve the production performance, meat quality, and reproductive performance of sheep [31,32]. Therefore, this study hypothesizes that supplementary feeding of Sewa sheep in the autumn can enhance their essential amino acid metabolism in the liver, which would be beneficial to their health.

Previous studies have indicated that the PI3K-Akt signaling pathway and the PPAR signaling pathway play crucial roles in animals’ adaptation to high-altitude environments [12,17]. The PPAR signaling pathway is essential for processes such as metabolism, fat breakdown, fat generation, and cell growth [33,34]. Some research has pointed out that under grazing conditions, the PPAR signaling pathway, associated with fat breakdown, is upregulated [35,36]. However, other studies have shown that in the cold season, compared to the warm season, there is a significant enrichment of DEGs in the PPAR signaling pathway in the liver tissue of grazing yaks, with downregulation of gene expression involved in fatty acid uptake (such as Lipase, Lipoprotein (*LPL*), Oxidized Low Density Lipoprotein Receptor 1 (*OLR1*)), fat generation (such as *SCD*, *FASN*, and Fatty Acid Desaturase 2 (*FADS2*)), fat breakdown (such as Fatty Acid Binding Protein 4, *FABP4*), the rate-limiting step of fatty acid uptake (such as *SLC27A2*), and fatty acid oxidation (such as *CPT1C*) [17]. In this study, compared to the summer, there was a significant enrichment of DEGs in the PPAR signaling pathway in the liver of Xizang sheep in autumn, with downregulation of gene expression involved in fat generation (such as Perilipin 1 (*PLIN1*), Acetyl-CoA Acyltransferase 1 (*ACAA1*), and *FADS2*), fat breakdown (such as Fatty Acid Binding Protein 1, *FABP1*), fatty acid transport (such as Sterol Carrier Protein 2, *SCP2*), and fatty acid oxidation (such as Acyl-CoA Oxidase 1 (*ACOX1*), Acyl-CoA Oxidase 2 (*ACOX2*), *ACSL1*, and Acyl-CoA Dehydrogenase, Long-Chain (*ACADL*)). This suggests that fatty acid synthesis in the livers of Xizang sheep during autumn grazing is suppressed, which is consistent with the research findings of Zheng et al. Furthermore, the PI3K-Akt signaling pathway plays a crucial role in regulating hepatic glucose homeostasis [37]. Activation of *PI3K* leads to the activation of *Akt* and the accelerated phosphorylation of *PDK1*, thereby inhibiting hepatic gluconeogenesis and promoting glycogen synthesis [37]. Previous research results have indicated that during the cold season, the PI3K-Akt signaling pathway is significantly inhibited, implying that the reduced expression levels of genes involved in this pathway might enhance hepatic glycogenolysis and gluconeogenesis to maintain the body’s energy balance [12,17]. Reports have indicated that compared to the Small-tailed Han sheep, Xizang sheep have a higher capacity for hepatic gluconeogenesis and ketogenesis under negative energy balance conditions, to cope with low energy intake and regulate whole-body energy balance in the harsh environment of the Qinghai-Tibet Plateau [38]. This study found that the expression of genes involved in the PI3K-Akt signaling pathway in the livers of Xizang sheep was downregulated, which is in line with the research results of Zheng and Jing et al. [17,38]. Therefore, this study speculated that Xizang sheep exhibit physiological characteristics of low fatty acid oxidation and lipolysis metabolism, as well as high gluconeogenesis, under autumn grazing conditions to maintain whole-body energy balance.

## 5. Conclusions

Through transcriptomics, this study explored the potential metabolic mechanisms by which Xizang sheep adapt to seasonal changes in the high-altitude environment under natural grazing conditions. Transcriptome analysis revealed that seasonal changes lead to differential alterations in various liver metabolic genes and related pathways, such as glycolysis or gluconeogenesis, fatty acid metabolism, and the degradation of valine, leucine, and isoleucine. Most importantly, the reduction in fatty acid synthesis, fatty acid oxidation, fat generation, and fat breakdown metabolism promotes gluconeogenesis by regulating the PPAR signaling pathway and the PI3K-Akt signaling pathway, thereby maintaining the whole-body energy homeostasis of Xizang sheep and facilitating their adaptation to the seasonal changes in the extreme high-altitude environment. This study not only lays the foundation for improving the health and resilience of Xizang sheep in high-altitude environments but also provides theoretical references for the production management of Xizang sheep.

## Figures and Tables

**Table 1:** Primer Sequences of DEGs.

Gene	Sense Primer	Anti-Sense Primer	Product Length (bp)	Annealing Temperature (°C)
*ENG*	CCGAGAGGTGCTGCTGATT	GCTGGTCCTTGGTGGTGAA	161	55.2
*B2M*	ACCTCTAAGCAGCACCATCA	AAGGCAGGCACAATGAACAA	171	54.25
*APP*	CGATGATGAGGAGGACGATGA	TACTGGCTGCTGTTGTAGGAA	161	54.95
*HGD*	GGAGCATGATGCCACAGTTG	TCACGGAACACCAGCAAGTA	194	55.05
*ACSM1*	TGGAGAAGGAGGGCAAGAGA	AGACACAAGCCACCACTCAG	200	55.45
*PHYH*	CGGATGCCAACTGTCACTAC	GACAAGAGTTTGAGCGAAGGG	193	54.8

**Table 2:** Significantly upregulated and downregulated top 10 genes.

Gene	*p* Value	log2FoldChange	Expression Trend
*RCAN1*	2.45754 × 10^21^	−4.75991038	Down
*STEAP4*	5.9405 × 10^−17^	−4.911815814	Down
*CTH*	4.41775 × 10^−15^	−3.150262917	Down
*SLC13A5*	3.58956 × 10^−14^	−2.734541083	Down
*ARG1*	3.64529 × 10^−14^	−2.344238661	Down
*BTG2*	3.88486 × 10^−14^	−4.823175073	Down
*LOC101107401*	4.61357 × 10^−14^	−2.387922887	Down
*ACMSD*	3.30911 × 10^−13^	−3.076393763	Down
*OAT*	5.35999 × 10^−13^	−4.000998993	Down
*SOCS3*	5.5126 × 10^−13^	−5.065369941	Down
*PAM*	1.01657 × 10^−13^	1.449317452	Up
*WNT2*	6.68279 × 10^−12^	2.811765971	Up
*LOC101105809*	1.13712 × 10^−11^	1.277836221	Up
*LDOC1*	1.44555 × 10^−10^	1.866047882	Up
*LOC101113217*	1.96848 × 10^−10^	6.360831156	Up
*GABRA3*	3.87898 × 10^−10^	3.971103718	Up
*CSRP1*	4.36592 × 10^−10^	1.595112982	Up
*COL14A1*	1.06764 × 10^−9^	1.912281876	Up
*CIART*	2.05887 × 10^−9^	2.414455512	Up
*novel.842*	4.21598 × 10^−9^	4.263920715	Up

## Data Availability

The original contributions presented in the study are included in the article, further inquiries can be directed to the corresponding author.
